# 

**Published:** 1901-11-02

**Authors:** 


### NOTICE TO CORRESPONDENTS.

All MSS., Letters, Books for Review, and other matters intended for the Editor should be addressed The Editor, "The Hospital," The Hospital Building, 28 & 29 Southampton Street, Strand, W.C.

The Editor cannot undertake to return rejected MS., even when accompanied by stamped directed envelope.

**Notice to Advertisers and Subscribers.**

All Advertisements, Orders for Copies of The Hospital, and Business Communications should be addressed to **THE MANAGER (Not to the Editor),** The Hospital, 28 & 29 Southampton Street, Strand, London, W.C.

The Cost of Subscription, post free, is as follows:—Per week, 2½d.; per quarter, 2s. 8d.; per half-year, 5s. 3d.; per annum, 10s. 6d. Foreign:—Per annum, 15s. 2d., payable, in all cases, in advance.

**NOTICE.—Vol. XXX. of "The Hospital" (April 1901, to September 1901), in handsome cloth binding will shortly be on sale at the office of this Journal, price, 7s. 6d. Cases for binding the Numbers contained in this Volume 1s. 6d. Index to Vol. XXX., 2½d., post free.**

**The Monthly Part for November is now ready. Price 6d., by post 8d.**

### BOOKS RECEIVED.

JOHN WRIGHT AND CO.

"Golden Rules for Diseases of Children." By George Carpenter, M.D.Lond., M.R.C.P. "'Golden Rules Series," No. xi.

BAILLIÈRE, TINDALL, AND COX.

"The Pocket Gray, or Anatomist's Vade Mecum." By the late Edward Cotterell, F.R.C.S. Fifth Edition, revised and edited by C. H. Fagge, M.B., M.S.Lond., F.R.C.S.

MESSRS. LONGMANS, GREEN AND CO.

"Diseases and Injuries of the Teeth." By Morton Smale, M.R.C.S., L.S.A., L.D.S., and J. F. Colyer, L.R.C.P., M.R.C.S., L.D.S. Second Edition.

### Scraps and Gleanings.

H.M. the Queen has consented to become the patroness of the St. Mary's Hospital for Sick Children, Plaistow.

The sum of £1,275 has been received during the past season from visitors for viewing Eaton Hall, and this sum will be given to charitable purposes.

The income raised during the year on behalf of Dr. Barnardo's Homes was £148,615, as against £147,094 in 1899; out of 90,675 donations 64,247 were in sums under £1 each.

The official returns show that during the month of September 147,967 refugees were sheltered and fed in the refugee camps in South Africa; during the month there were 2,712 deaths.

The North Eastern Hospital for Children has received a further instalment of £200 from the editor of *Little Folks* towards the endowment of the "Little Folks" ward in the new building about to be erected.

The Dumb Friends' League is appealing to the public for contributions of cast-off clothing, household articles, and other things to be disposed of at a large sale to be held on behalf of the League early in November.

The British and Foreign Sailors' Society have received from Mrs. Sam Lewis £1,000, being the cost of the site, on which will be erected, by permission of her Majesty, the "Alexandra Wing" of the Passmore Edwards Sailors' Palace.

The new General Hospital which is being erected at Stobhill by the Glasgow Parish Council will provide accommodation for 1,500 patients and 150 nurses with other members of the staff. Its total estimated cost is £200,000.

The Registrar-General's return for London for the week ending October 12th shows that, allowing for the increase of population, the births registered were 83 and the deaths 166 below the average numbers in the corresponding weeks of the last 10 years.

Since the foundation of the Domestic Servants' Benevolent Institution 375 pensioners have been elected, 95 at the present time receiving £15 to £20 per annum. The Society is appealing for further help and asks also for more publicity in making this organisation known.

### The Student of Medicine.

### APPOINTMENTS.

T. Barrowman, M.B., C.M.Glas., appointed District Medical Officer of the Grimsby Union. **A. J. Bennetts**, M.R.C.S.Eng., L.R.C.P.Lond., appointed Assistant Medical Superintendent of the Camberwell Infirmary, and Assistant Medical Officer of the Gordon Road Workhouse. **Arthur Cyril Bird**, M.R.C.S.Eng., L.R.C.P.Lond., appointed Honorary Surgeon at the Victoria Cottage Hospital, Sidmouth, Devon. **Albert Henry Bygott**, M.B.Lond., appointed District Medical Officer and Public Vaccinator for the Dereland District of the Aston Union. **W. L. Byham**, L.R.C.P., L.R.C.S.Edin., appointed District Medical Officer of the Staines Union. **E. Treacher Collins**, F.R.C.S.Eng., appointed Ophthalmic Surgeon to the Charing Cross Hospital and Lecturer on Ophthalmology at the Medical School. **Mary Stewart Deacon**, M.B., B.S.Lond., L.R.C.P.&S.Edin., appointed Medical Officer of Health to the Accra Town Council, West Africa. **William Fordyce**, M.D.Edin., appointed Assistant Physician to the Edinburgh Royal Maternity and Simpson Memorial Hospital. **C. Hadley**, M.R.C.S.Eng., L.R.C.P., appointed District Medical Officer of the Nuneaton Union. **Oakley Elford Higgens**, M.A.Cantab, M.B.Dunelm., M.R.C.S., L.R.C.P., appointed Honorary Medical Officer to the Holloway and N. Islington Dispensary. **Miss M. Joyce**, B.S.Durh., appointed Assistant Medical Officer to the Camberwell Infirmary. **E. M. Knowling**, M.B.Camb., M.R.C.S.Eng., appointed District Medical Officer of the Pembroke Union. **W. Langran**, L.R.C.P., L.R.C.S., appointed Medical Officer of Health for the Axminster Rural District. **A Miller**, M.B., B.C.Camb., appointed District Medical Officer of the Grimsby Union. **John Herbert Parsons**, M.B., B.S., B.Sc., F.R.C.S., appointed Curator and Librarian to the Royal London Ophthalmic Hospital, City Road. **G. A. Robinson**, M.B., B.S.Lond., M.R.C.S.Eng., appointed Surgeon to the Samaritan Hospital, and Lecturer in University College, Nottingham. **J. A. Stephen**, M.A., M.B., Ch.B., D.P.H.Aberd., appointed Police Surgeon and Prison Surgeon Elgin County, also Medical Officer of Health for Burgh of Branderburgh and Lossiemouth.

